# A simulation model of African *Anopheles *ecology and population dynamics for the analysis of malaria transmission

**DOI:** 10.1186/1475-2875-3-29

**Published:** 2004-07-30

**Authors:** Jean-Marc O Depinay, Charles M Mbogo, Gerry Killeen, Bart Knols, John Beier, John Carlson, Jonathan Dushoff, Peter Billingsley, Henry Mwambi, John Githure, Abdoulaye M Toure, F Ellis McKenzie

**Affiliations:** 1 Fogarty International Center, National Institutes of Health, 16 Center Drive, Bethesda MD 20892, USA; 2 Kenya Medical Research Institute, Centre for Geographic Medicine Research – Coast, P.O. Box 428, Kilifi, Kenya; 3 International Centre of Insect Physiology and Ecology, P.O. Box 30772, Nairobi, Kenya; 4 Ifakara Health Research and Development Centre, PO Box 53 Ifakara, Kilombero District, Tanzania; 5 Entomology Unit, FAO/IAEA Agriculture and Biotechnology Laboratory, A-2444 Seibersdorf, Austria; 6 Global Public Health Program, University of Miami, South Campus, 12500 SW 152nd Street, Building B, Miami, FL 33177, USA; 7 Tulane University, New Orleans, LA 70118, USA; 8 University of Aberdeen, Zoology Building, University of Aberdeen, Aberdeen AB24 2TZ, UK; 9 School of Mathematics, Statistics and IT, University of Natal, Private Bag X01 Scottsville, 3209 Pietermaritzburg, South Africa; 10 Faculty of Medicine, Pharmacy, and Dentistry, Malaria Research and Training Center; B.P. 1805 Bamako, Mali

## Abstract

**Background:**

Malaria is one of the oldest and deadliest infectious diseases in humans. Many mathematical models of malaria have been developed during the past century, and applied to potential interventions. However, malaria remains uncontrolled and is increasing in many areas, as are vector and parasite resistance to insecticides and drugs.

**Methods:**

This study presents a simulation model of African malaria vectors. This individual-based model incorporates current knowledge of the mechanisms underlying *Anopheles *population dynamics and their relations to the environment. One of its main strengths is that it is based on both biological and environmental variables.

**Results:**

The model made it possible to structure existing knowledge, assembled in a comprehensive review of the literature, and also pointed out important aspects of basic *Anopheles *biology about which knowledge is lacking. One simulation showed several patterns similar to those seen in the field, and made it possible to examine different analyses and hypotheses for these patterns; sensitivity analyses on temperature, moisture, predation and preliminary investigations of nutrient competition were also conducted.

**Conclusions:**

Although based on some mathematical formulae and parameters, this new tool has been developed in order to be as explicit as possible, transparent in use, close to reality and amenable to direct use by field workers. It allows a better understanding of the mechanisms underlying *Anopheles *population dynamics in general and also a better understanding of the dynamics in specific local geographic environments. It points out many important areas for new investigations that will be critical to effective, efficient, sustainable interventions.

## Background

Not so long ago, in 1998, Sherman declared: "Of all the human afflictions, the greatest toll has been exacted by malaria. Even today, malaria, which is caused by protozoan parasites of the genus *Plasmodium*, disables and kills more people than any other infectious disease." [1]

In line with the pioneering models of Ross (1911) and Macdonald (1957), malaria interventions such as breeding-site reduction and insecticide use have been considered the most effective and practical ones for reducing malaria transmission. Bednets and house screening serve as personal protection, and bednet-associated effects on malaria prevalence appear to be greater than can be accounted for by personal protection [2]. These interventions have produced good results, but in much of the world malaria remains uncontrolled. Furthermore, malaria vectors are increasingly developing insecticide resistance. At every level of research, policy and practice, malaria control can be helped by models that are both more comprehensive and closer to the day-to-day realities of malaria (K. Dietz in [3]). As Bradley (1982) has pointed out, "for real progress, the mathematical modeller, as well as the epidemiologist, must have mud on his boots." The aim of this study is to provide a framework and a tool for modelers to work closely with field workers in malariology, particularly entomologists.

The study also aims to achieve a broader analysis and deeper understanding of the complex mechanisms involved in malaria transmission, in order to aid intervention programs. The idea of controlling malaria through the introduction of genetically modified mosquitoes is gaining increasing attention, for instance, but will first need to be tested critically, in trials that will necessarily involve models.

Thus the work presented below represents only a beginning, and it has two major aims. First, it introduces an approach to help researchers account for ecological variables that are key determinants of malaria vector population dynamics. When fully calibrated, this approach will provide an integrated platform for hypothesis testing with complex temporal and spatial data; ultimately, it should help by providing forecasting capabilities.

Of perhaps even greater importance, this first model provides a vehicle for assembling and structuring existing knowledge, thereby pointing out critical areas in which knowledge is lacking and very much needed. Thus it is a means of identifying and organizing important research priorities and indicating their epidemiological implications.

One of the most important strengths of this model is to combine biological and environmental variables. As stated by [4], the combination of intrinsic and extrinsic determinants of mosquito-borne disease incidence should be the focus of future research. This is critical both in controlling these diseases and reducing the severity of epidemics by predicting them.

Approximately 70 species of *Anopheles *have been implicated in malaria transmission worldwide. In Africa the major vectors are *Anopheles gambiae *sensu lato, which is considered the most important in most regions, *Anopheles arabiensis*, which is part of the preceding complex but with distinct characteristics, and *Anopheles funestus*, which is often reported as the second most important species in terms of malaria transmission and, more particularly, is considered the end-of-rainy-season vector that sustains the parasite. This work focuses on the major vector in sub-Saharan Africa *An. gambiae*, but much of what follows may be applicable to *An. arabiensis*, and even to *An. funestus *separately and all together, with inter-as well as intra-species competition.

This paper describes the first model of malaria vector population dynamics integrating both biological and environmental factors.

## Methods

The model incorporates basic biological requirements for *Anopheles *development on an individual basis and, using local environmental data as input, allows the simulation of the aggregate dynamics of *Anopheles *populations. The life cycle of each individual proceeds through four stages: three immature stages, which occur in a water body – egg, larva, pupa – and then the mature stage, a flying adult. An adult female disperses from the natal water body and begins a cycle which is maintained throughout the rest of life-alternating between obtaining a bloodmeal and ovipositing in a water body.

**Figure 1:**
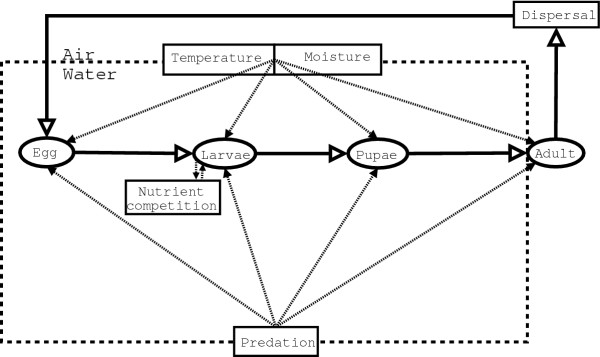
Model description.

Five major factors are considered here as characterizing *Anopheles *population dynamics, by means of mechanisms detailed below (see figure 1 for a schematic):

**Temperature **is a critical regulator of growth and development within each stage, in determining the end of one stage and the beginning of the next and in regulating the length of the gonotrophic cyle.

**Moisture**, in the form of precipitation and relative humidity, is a second key abiotic factor, with effects that in part interact with those of temperature.

**Nutrient competition **is a major potential regulator which is considered to induce mortality in the larval stage. In addition, there is a minimum weight requirement for the transition from larva to pupa, and, through its influence on adult weight, the relation of larval weight to fecundity.

**Predation and Disease**, in which pathogens are included, is a second important mortality-inducing factor, which is considered in local terms relative to the water body.

**Dispersal**, or the adult female's movement in space, is a critical factor in the cycle of seeking blood meals and oviposition sites. The model explicitly represents spatial locations of individual adults, though it does not fully engage this capacity in the analyses presented here.

The model is implemented as a software package in the C++ object-oriented programming language, in the Microsoft Windows 98 operating system, and is available from the corresponding author upon request. It was developed and run on a personal computer with a Pentium 3 processor 933 MHz and a relatively small memory of 256 Mb.

### Temperature

Because malaria vectors are poikilothermic, temperature is a critical variable in malaria epidemiology. For instance, in the range of 18°C to 26°C, a change of only 1°C in temperature can change a mosquito's life span by more than a week [5].

Here, in line with the work of Focks et al. [6] on *Aedes aegypti*, the enzyme kinetics model derived by Sharpe and DeMichele [7] is used, based on absolute reaction rate kinetics of enzymes for the temperature-dependent developmental rates of eggs, larvae and pupae and the duration of the gonotrophic cycle, in the simplified form derived by Schoofield et al. [8].

This equation is derived on the basic assumption that poikilotherm development is regulated by a single control enzyme whose reaction rate determines the development rate of the organism [7,8]. This is of special interest because each parameter of the equation has a biological significance that may have an epidemiologic impact.

At time step *t*_*n *_of *t*_0_, *t*_1_, ..., *t*_*n*_, the development within each of the four stages, during the time step *Δt*_*k *_= *t*_*k *_- *t*_*k*-1_, is defined by:

*d*_*k *_= *r*(*T*_*tk*_)·*Δt*_*k*_.     (1)


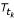
 is the mean temperature (°K) over the time interval *k *and *r*(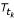) the developmental rate per hour at temperature *T*(°K), given by the following equation:


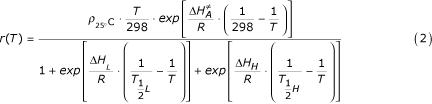


where *ρ*_25°_C is the development rate per hour at 25°C, under the assumption that there is no temperature inactivation of the critical enzyme; 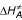 is the enthalpy of activation of the reaction catalyzed by the enzyme (*cal·mol*^-1^); *ΔH*_*L *_is the enthalpy change associated with low temperature inactivation of the enzyme (*cal·mol*^-1^); 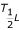 is the temperature (°K) where 50% of the enzyme is inactivated by low temperature; *ΔH*_*H *_is the enthalpy change associated with high temperature inactivation of the enzyme *(cal·mol*^-1^); 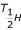 is the temperature (°K) where 50% of the enzyme is inactivated by high temperature; and *R *is the universal gas constant (1.987*cal·mol*^-1^).

The cumulative development, depending only on temperature at each time step *t*_*n*_, of each of the three stages (egg, larvae, pupae) and the length of the adult gonotrophic cycle is defined as:


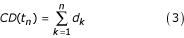


with *d*_*k *_defined above in equation 1.

As detailed below, other factors are also considered, including a particular case for the larval stage that takes food requirements into account.

Variability is allowed for in the cumulative development time, *CD*(*t*_*n*_), with a default value of 10% and a stage is considered completed, such that the next stage begins when:

*CD(t) *>*CD*_*f *_= 1 + *G*(0,0.l)     (4)

where *G *is a normal random variable.

A survey of the literature reveals how very little developmental-rate data is available for *Anopheles*, even for the most important African malaria vectors. The deficit is striking for all of the three major malaria vector species in Africa. We have fit the curve defined by equation 3 to all of the relevant published data. Those data are compiled in tables 1 and 2, for *An. gambiae *sensus lato.

One reference provided only the total *An. gambiae *development time from egg to adult [5], we have then estimated the development time for each of the three constituent stages in according with the other data, and also assumed longer development times at low temperatures.

The only gonotrophic cycle data available in relation to temperature was for *An. arabiensis*, part of the *An. gambiae *complex.

**Figure 2:**
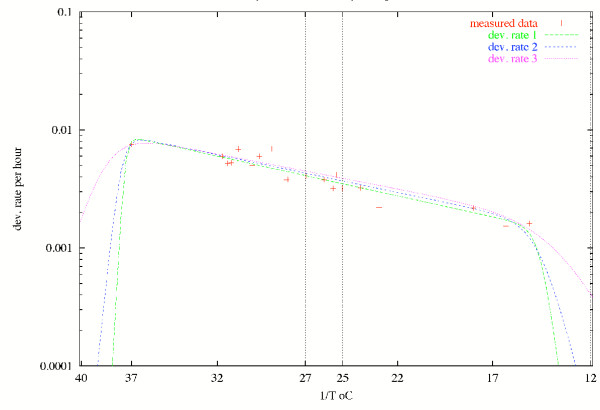
Three possible curves fit to *An. gambiae *larvae development rate data.

**Figure 3:**
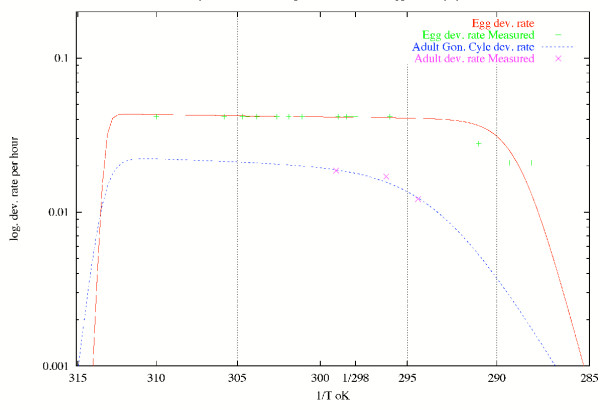
Egg and adult development rates.

**Figure 4:**
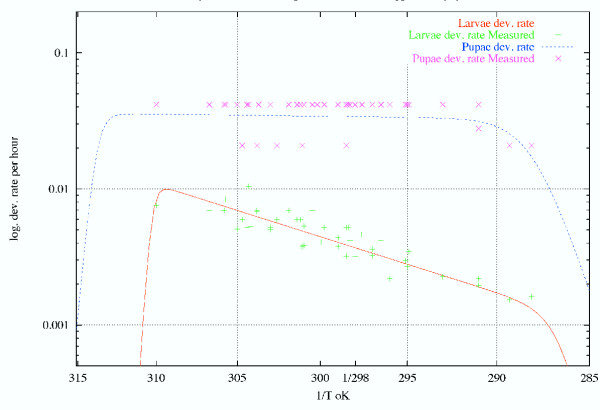
Larvae and pupae development rates.

All three curves shown in figure 2, for different parameters of equation 2, provide similar fits to the *An. gambiae *data in tables 1 and 2. These different curves have important implications for vector population dynamics and reinforce the need for more data for these species, particularly at the temperature extremes (low and high), in order to fit an optimal curve. Until there is data for the extreme temperatures, any number of curves might fit the data. Three such curves are illustrated in figure 2. For the purposes of this paper the middle of these three curves has been chosen, with parameters shown in table 3. The curves for all four stages are shown in figures 3 and 4, with parameters in table 3.

*An. gambiae *females are one-day old when they take their first blood meal, according to [9]. This greater length of the first gonotrophic cycle has been taken into account [9][10] by defining a coefficient *U*_*FirstGon *_which represents the time lag before the first blood meal expressed as a percentage of the gonotrophic cycle length. Therefore, the first gonotrophic cycle is considered completed if:

*CD(t) *>*CD*_*f *_= 1 + *U*_*FirstGon *_+ *G*(0,0.1)     (5)

*U*_*FirstGon *_has been set to 0.5 for *An. gambiae. *All subsequent gonotrophic cycles follow equation 4.

#### Thermal mortality

Although the range of variation of water temperature is very wide, it is rarely taken into account in the literature. Some authors have recorded temperatures close to 40°C in small pools [5,11,12]. Such temperatures exceed the thermal death point of many species, including *An. funestus *[5,12]; this may help to explain why these species are rarely found in small pools. Based on these observations [5,12], a daily mortality in the larval stage of 10%, 50% and 100% for a maximum water temperature of 1, 2 and 3°C above the thermal death point, respectively, has been considered. According to [5] the thermal death point for *An. gambiae *is set to 40°C.

### Moisture

*Anopheles *usually develop in natural water bodies, such as puddles, pools or streams [11-14]. The model must take into account two critical parameters in a water body, the temperature and the volume of water. In this stage of the project it was not possible to develop a full water-balance model to estimate those parameters but it should be possible in the future.

Cloud coverage is likely to be relatively important because of its impact on the water temperature, but this variable is rarely available in climate data. However, it is known that a relative humidity of 100% is usually associated with complete cloud coverage and rain and a relative humidity less than 50% with dryness and almost no clouds. Hence an estimate of cloud coverage as a function of relative humidity *RH *was made. A clear sky, without clouds (0), for relative humidity below 50%, linearly increases to completely cloudy (1) for relative humidity above 95%, as follows:


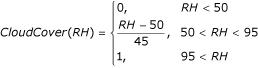


The maximum water temperature of a water body depends on the cloud coverage and a user-defined coefficient *U*_*SunExpo *_that describes the water body's sun exposure. This user-defined coefficient represents the coverage or shaded percentage of the particular water body, ranging from 0 for complete shade to 1 for complete sunlight exposure. By default it is set to 1.

If the maximum air temperature in degrees Celsius is *T*^*M*^, it is estimated that the maximum water temperature 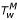 in accord with the water volume *x *(in liters) is 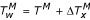, where:


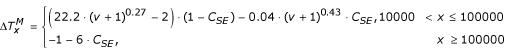


with *C*_*SE *_= *U*_*SunExpo*_*·CloudCover*(*RH*). The minimum water temperature is taken as the minimum air temperature.

The following formula estimates the daily dynamics of water height *W*_*H *_in a water body:


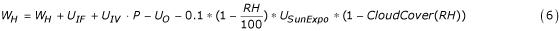


where *U*_*IF *_is the fixed daily water intake in *mm·day*^-1 ^(e.g. from a stream, pipeline, human activity, etc.); its default value is 0. *U*_*IV*_, the variable daily water intake in *mm·day*^-1^is set in accord with the precipitation and the surrounding area's topology. Its default value is 1, which would apply to a water body in a flat area, such that only direct rainfall fills the water body. The user can set a particular value: for a water body on a slope, this coefficient should reflect the volume of water intake given 1 *ml *of precipitation in the area. *P *is the precipitation in *mm *per day, and *R*_*H *_is the relative humidity. *U*_*O*_, in *mm·day*^-1^, is the daily loss of water due to soil infiltration and evapotranspiration. By default, this parameter is set to a mean value of 3 *mm·day*^-1^.

The water bodies are approximated by means of simple geometric objects, such as cubes and cylinders. The default geometric object is a box; its dimensions (length, width, depth) can be entered by the user. Therefore, the volume of water available in the water body is calculated from the particular shape of the water body and the water height calculated above (equation 6).

#### Aestivation and diapause

Unlike the eggs of *Aedes aegypti*, which, it has been shown, can survive in dry soil for more than two months [6], recent work [15] indicates that *Anopheles *eggs cannot survive more than 15 days on dry soil. Thus, since some African regions with endemic malaria experience drought periods longer than two months, the only plausible alternative seems to be adult aestivation. This is another aspect of *Anopheles *biology in which much more data is needed. The different survival probability during aestivation has been arbitrarily set as shown in table 4.

Aestivation or diapause is triggered by the non-availability of water (when water bodies are completely dry) for all stages. For the adult stage, aestivation is also triggered by a relative humidity arbitrarily chosen here at less than 40%, though even this may prove to be high in some area.

### Nutrient competition

Some combination of regulatory mechanisms limits the size of any population of any species. The most important, for many species, can be described as density-dependent regulation, or competition for space and/or food, which is assumed to summarize or integrate complex, difficult-to-measure mechanisms, such as food mass conversion. For the sake of simplicity and practicality, the basic ecological concept of carrying capacity [16] has been used here. This concept has been applied primarily to the larval stage since it is the longest immature stage and is the only immature stage in which the mosquitoes feed and is, therefore, likely to be the most sensitive to competition.

For each water body *i *a carrying capacity *K*(*i*) (in *mg*) has been defined as:

*K*(*i*) = *L*_*Max*_·*S*(*i*)·*U*_*Carrying *_    (7)

where *L*_*Max *_is the maximum larval biomass density, defined for all species *j *by:


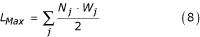


where *N*_*j *_is the larval population size per surface unit (*m*^2^) for species *j*, and *W*_*j *_is the approximate mean weight of species 
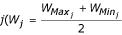
, with 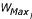 and 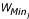 being the maximum and minimum possible weight in species *j*, respectively), divided by 2 in equation 8 to correct for the greater size of the low-weight larval population. *L*_*Max *_= 300 *mg*·*m*^-2 ^has been arbitrarily set for larvae. *S*(*i*) is the available water surface in water body *i*, and *U*_*Carrying *_is a positive user-defined coefficient for each water body, to correct for particular water-body characteristics; by default it is set to 1. Thus, for each water body at peak season periods, the maximum larval biomass density *L*_*Max *_is estimated by measuring the larval population size at its maximum.

#### Density-dependent mortality

Resource competition is considered as a cause of mosquito mortality only for the larval stage. For species *j *[16] the natural increase of the total larval population size, *N*, (without mortality) can be defined by:


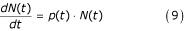


where *p *is the proportion of larvae that is newly-hatched eggs, estimated by:


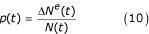


where *ΔN*^*e*^(*t*) is the number of individual eggs entering the larval stage.

The carrying capacity *K*(*i*) of a particular water body i is defined above (Equation 7). In general, the larval population increase is given by:


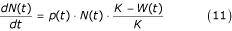


where *W*(*t*) is the current larval biomass overall (in contrast to *W*_*j*_, the approximate mean weight of species *j*; see equation 8).

The larval per capita density-dependent mortality rate *m *for all species can be approximated by:


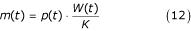


#### Weight

As noted above, the larval stage is the only immature stage with food intake and, therefore, with weight changes. Thus, this stage is the key determinant of the final adult weight.


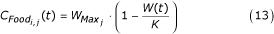


where 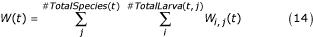


and 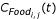 is a coefficient that describes food availability for an individual *i *of species *j*, 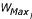 is the maximum possible weight for species *j, W*(*t*) is the current larval biomass, *K *is the carrying capacity of the water body, and *W*_*i*, *j*_(*t*) the weight of individual *i *of species *j *at time *t*. For each time step *k*, for species *j*, the weight of individual *i *increases linearly as 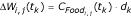, where *d*_*k *_is the thermal development in time period *k *(equation 2). The weight in the larval stage is then calculated as:


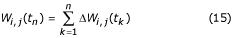


This formula allows the individual larva to have a maximum weight in accord with its species 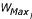 when the larval biomass *W << K*. At the other extreme the weight increase will be almost zero if *W *≈ *K*. Note that this formula allows both intra-and inter-species competition for food.

From [5,17-19] the weight parameters for each species have been set as shown in table 5.

For the purpose of stochastic simulation variability has been allowed, again with a default value of 10%, as follows:

*W*_*i*, *j *_= *W*_*i*, *j *_+ *G*(0, 0.1)     (16)

where *G *is a normal random variable. The larval stage is regarded as completed, such that the pupa stage begins, when the thermal development *CD *is completed (Eq. 4) and *Weight *>*Weight*_*Min*_.

The relative weight of an individual within its species is used as an important factor in subsequent subsections on fecundity and number of blood meals, in which the following coefficient is used:


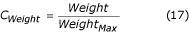


### Predation and Disease

Predators and pathogens are an important regulating factor and are sometimes reported to be the major cause of mortality [20].

#### Egg

Little has been reported about *An. gambiae *egg mortality, from predation or any other cause, beyond an observation (Beier, personal observation) that up to 83% of eggs hatch after one day of drying on sandy loam soil. Without more information, the total egg mortality for each species was arbitrarily set at 5% as a fixed pre-development mortality for the overall batch and a daily survivorship of 0.99.

#### Larvae and Pupae

Service [20] points out that *An. gambiae *population sizes rise to a peak just after a drought period and then decrease to a roughly stationary level. Life cycles of predators on immature *An. gambiae *are generally longer than those of their prey, and during the latter phases predators are found in non-predatory stages (i.e. not preying on immature *An. gambiae) *[20]. Intensity of predation appears to be highly related to the early peak in prey, but there is still a regulatory effect even in the absence of predators. Hence, it is likely that predation is not the only major cause of mosquito mortality [20].

Service [20] evaluated immature *An. gambiae *sensu lato mortality from predation in two experiments, one in which predator density was high and another in which spraying had reduced predator density. His results are summarized in table 6. With respect to pathogens and parasites, he found that 2.1% to 15.9% of *An. gambiae *were infected.

Active predation exhibits a lag time around the mean life-cycle length of the prey [20]. During the lag period *l*, if *t = *0 is the start of this period, a curve should show a gradual increase in predation.

**Figure 5:**
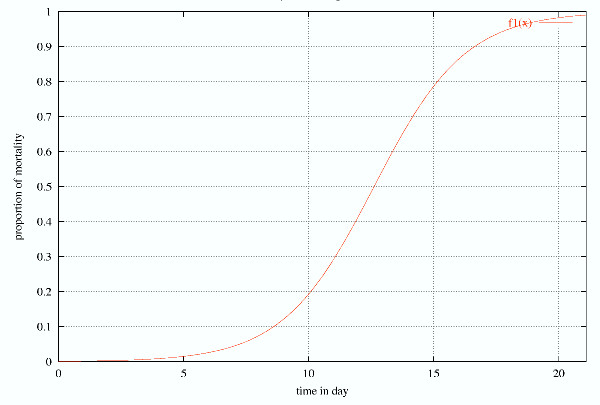
Predation percentage function of time (lag time).

The conditions leading to a new predator lag period could occur, for instance, when a dry water body gains water or after a control intervention killing the predators. If (fig. 5):


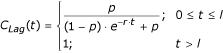


with 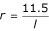 and *p *= 0.001, then the total larval and pupal mortality due to predators and pathogens for species *j*, can be expressed as:


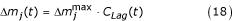


Note that *Δm*_*j*_(*t*) differs from *m*(*t*) in equation 12, which represents density-dependent mortality. For all species *j *the following were arbitrarily set: 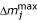 = 25% for larvae and 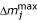 = 10% for pupae. 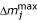 = 25% is converted to a daily mortality rate as:


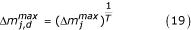


where *T *is the individual's developmental time. Thus at *t *= 0, the beginning of the lag period, *Δm*_*j*_(*t*) ≈ 0, and at *t *≥ *l*, 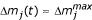 for species *j*.

On adding to the density-dependent mortality *m*_*j *_the mortality due to predation and pathogens *Δm*_*j*_(*t*), for each species *j*, we obtain a new equilibrium *K*_*p *_<*K*, given *K *in equation 11, where


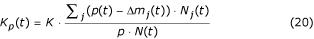


where *N*_*j *_is the larval population size for species *j (N*(*t*) = 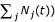.

#### Adult

There are several published studies of adult mortality rates [9,21] for *An. gambiae *and *An. funestus. *The causal mechanisms are not clear, but some authors report adult predators preying on adult mosquitoes at oviposition sites [20]. It is assumed that predation-related adult mortality is focused at the water body and that survivorship is greater with fewer predators present.

Oviposition typically occurs every two to three days (see above). Accounting for the low predation during the previously-defined predator lag time, the daily adult survival probability is taken to be 0.911 for a non-ovipositing day and 0.911 - 0.1·*C*_*Lag*_(*t*) for *An. gambiae *sensus lato.

### Dispersal

The mechanisms governing mosquito dispersal in general remain unknown. Wind strength and direction are likely to be important factors, for instance, but relevant data are rarely reported. Very little is known about the relative attractiveness of individual humans and individual water bodies to *Anopheles*, but these cues, along with distance, must be key factors in dispersal.

In most tropical regions, bloodmeals are taken at night, between 6:00 pm and 6:00 am. As the mosquitoes are active during the night, for simplicity bites were modelled only in houses. Bloodmeal source selection is modelled by a two-step process, first a choice of house and second a choice of individual human within the house. Anthropophily, the proportion of bites taken on humans, can be set for each *Anopheles *species overall; the default value of this parameter is 1. Exophily is expressed as the proportion of fed mosquitoes that leave the house during the first half of the gonotrophic cycle. For *An. gambiae *the default value of this parameter is 75%.

The model explicitly, dynamically represents individual locations in space, but at this stage the adult female alternately chooses at random among some number of water bodies for an oviposition site, and at random among some number of houses and individuals within the chosen house, for a bloodmeal. That is, the choices do not reflect relative distance, attractiveness, wind or other features the model is designed to address in future phases of development.

#### Multiple bloodmeals and multiple bites

In addition to the greater length of the first gonotrophic cycle (Equation 5), Brengues [9] has shown that, to complete their first gonotrophic cycle, 42% of female *An. gambiae *and 63% of female *An. funestus *require a second bloodmeal one day after the first one. Here the probability of having a second bloodmeal within the first gonotrophic cycle is related to the weight of the individuals: there is a second bloodmeal when the coefficient *C*_*weight *_is less than 0.4 for *An. gambiae.*

For multiparous females, there is a second bloodmeal when *C*_*weight *_is less than 0.1.

According to [22], 14% of female *An. funestus *and 19% of female *An. gambiae *that had just fed had taken only a partial bloodmeal. These figures are used to represent the proportion of females that take a subsequent bite within what is considered the same bloodmeal.

#### Fecundity

The number of eggs oviposited by individuals shows a wide range of variation, both within and between experiments [17,18,23,24]. The mean number of eggs oviposited is defined by *m = *100, with a standard deviation *s *= 50. In the absence of more precise information these values are assumed. The number of eggs oviposited is simulated as:

*N *= *G(m, s)*·*U*_*Egg *_    (21)

where *U*_*Egg *_is a positive user-defined coefficient set to fit local observations, by default set to 1, and *G *is a normal random variable. Because fecundity is closely tied to body size, a variability of 50% of the number of eggs is allowed as a function of the individual's weight, as follows (see [18][23]):

*N*' = *N*·(0.5 + 0.5·*C*_*weight*_)     (22)

The male-female ratio at emergence from the pupa stage is assumed to be 1:1.

## Results

**Figure 6:**
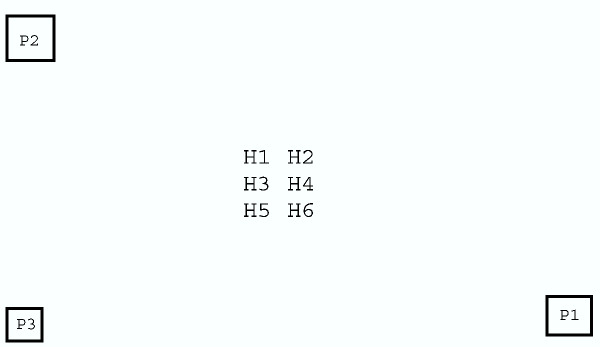
Example schematic.

**Figure 7:**
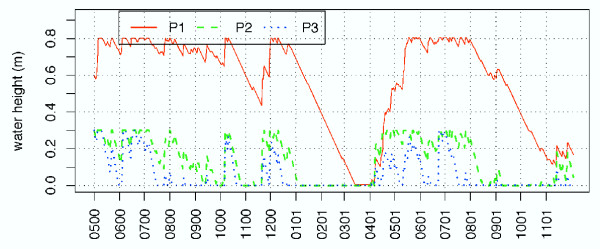
Water height in pools.

**Figure 8:**
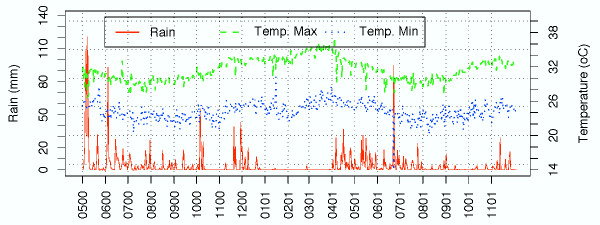
Rainfall and Temperature

**Figure 9:**
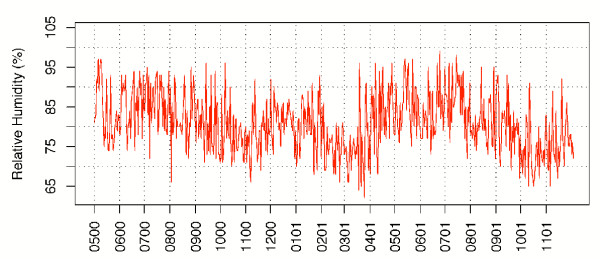
Relative humidity

A simple example is used to show how the model can help to achieve a better understanding of vector population dynamics and determine key underlying factors. In particular, the influence of temperature, moisture, predation and nutrient competition on adult abundance is investigated. The example is taken as a small cluster of six houses, each with five residents, and a total of three oviposition sites (figure 6 and table 7. An attempt has been made to reproduce some important characteristics of a local environment by considering two types of pools: a semi-permanent pool, P1, and two temporary pools, P2 and P3 (see figure 7 and table 7. As noted above, at this stage each mosquito in the model chooses at random among oviposition sites and among houses and residents at the appropriate points in her gonotrophic cycle. Temperature and moisture inputs were obtained based on data from Kilifi, on the coast of Kenya. Figures 8 and 9 show daily precipitation, minimum and maximum temperature and relative humidity reported there over the 20 months from May 1, 2000 to December 31, 2001. In this region there are two primary rainy seasons: April-June and October-November. Except where noted, the default values were used for parameters, as given above.

### Effects of temperature

**Figure 10:**
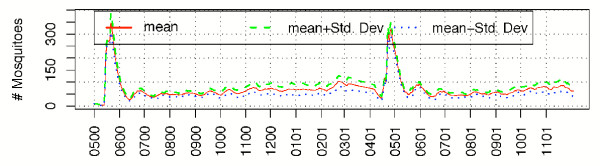
Simulated *An. gambiae *adult abundance at actual temperatures for 20 simulations

**Figure 11:**
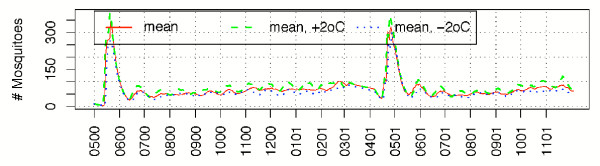
*An. gambiae *adult abundance, mean of 20 simulations for each temperature level

In the first set of simulations there are 300 eggs and 10 adults, with all six houses but only pool P1 present. Figure 10 shows the variability and mean of twenty replicates realizations of the simulation model, an effect of the stochasticity allowed in the cumulative development time (equation 4), length of initial gonotrophic cycle (equation 5) and number of eggs oviposited (equation 21). The abundance curve is predicted from the preceding environmental data, with each run started on May 1, 2000. This *An. gambiae *adult mean curve shows similarities to several published curves, at much wider scales [25], in that there are relatively low levels of mosquitoes throughout the year, with fluctuations in abundance that may correspond to the limitations of competition and/or predation and several very high peaks in short time intervals. To analyse the effects of temperature, two additional temperature curves were used, one in which the actual temperatures are increased by two degrees and one in which they are lowered by two degrees Celsius, the results are shown in figure 11. Table 8 shows the impact of temperature on adult abundance. For *An. gambiae *(figure 11), with increasing temperature there is a general increase in the level and number of peaks. As detailed above in the section on Temperature (table 1 et seq.), the egg-to-adult development time is shortened with higher temperature, thus producing more mosquitoes. The two-degree temperature rise increases *An. gambiae *adult abundance over the full 19 months by 15%; the two-degree temperature drop decreases it by 17% overall. Recall that multiple factors interact to determine the adult abundance at each point; however, predation is probably not a critical biotic regulating factor by the time of the initial peak, for instance, but nutrient competition/carrying capacity probably can have a strong impact at late stages of this initial peak.

In general, although the drought period from March 12, 2001 to March 31, 2001 has the effect of allowing a first big peak in adult abundance for *An. gambiae*, it also synchronizes the first peak, and might be important for control intervention purposes.

The overall pattern of adult abundance appears well-conserved, and the variability relatively minor.

However, as noted above, the aim here is simply to suggest the potential of the model. Figure 10 shows the standard deviation (variability) of the twenty replicate for each date.

### Effects of temporary pools

**Figure 12:**
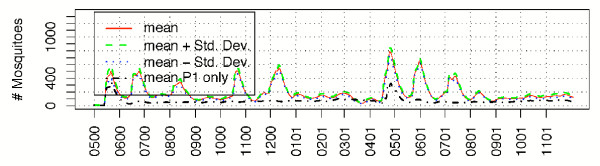
*An. gambiae *adult abundance with all three pools (P1, P2 and P3) mean of 20 simulations

Here *An. gambiae *is considered and examined for the effect on adult abundance of adding pools P2 and P3 to the semi-permanent pool P1, beginning with 10 adults and 300 eggs in each pool. Pools P2 and P3 may be classified as temporary, since they dry two or three times during the year (see figure 7). Beside the expected increase in the total number, there is a much more dramatic fluctuation in the mosquito abundance curve, with six added major peaks (figure 12).

### Effects of interventions

Here *An. gambiae *is considered, with pool P1 only, and show how the model might be of help in reducing peaks in adult abundance by helping to optimize the control of larval and adult populations. Recall that the goal here is not to allege or prove a particular finding, which can depend on a specific environmental situation, but to show how the model could help address a given question in a specific environmental situation, and help in understanding the mechanisms involved. The aim is to show examples, with graphical representation, of how such a model can be a powerful tool in research on malaria vector dynamics. For the purpose of the first analysis the predator population is excluded from any effects of the larval control intervention. Therefore, the impact of the predator as described above (in the Predation section) will remain constant.

Although the focus is the first major peak in adult abundance, the analysis could be transposed to any period. Interventions that take effect in two periods are compared, the first beginning on May 6, 2000, at the beginning of the first major peak, and the second beginning 15 days later, on May 21, 2000. A successful one-time larval control intervention is simulated by imposing 80% mortality on all larvae present during 10 consecutive days. An adult control intervention that consists of spraying surfaces inside houses with residual insecticide is simulated by imposing 75% mortality on blood feeding adults during a 25-day period.

**Figure 13:**
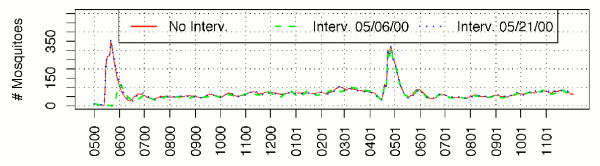
*An. gambiae *adult abundance with larval control intervention

Figure 13 indicates that the later larval-control intervention (5/21/00), though done at the highest adult abundance rates, would have almost no effect on overall adult abundance, since it happens at a period of lower larval abundance. Still worse, it could lead to the production of bigger mosquitoes by diminishing the nutrient competition. On the other hand, a larval-control intervention that began only 15 days earlier would nearly eliminate the entire first peak in adult abundance. This emphasizes the need of good forecasting tools.

**Figure 14:**
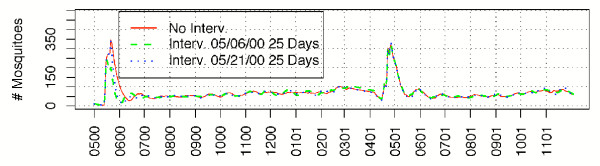
*An. gambiae *adult abundance with adult control intervention

**Figure 15:**
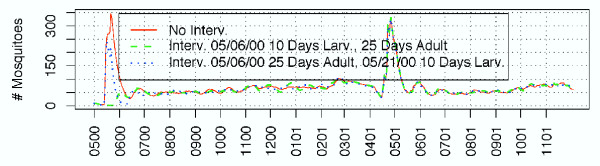
*An. gambiae *adult abundance with larval and adult control intervention

Similarly, for an adult-control effort (figure 14), the later control intervention would have very little impact, but the first peak in adult abundance could be decreased consequently by an effort that began only 15 days earlier. At this stage the model does not take into account such important factors as insecticide resistance and mosquito avoidance behavior, which would tend to diminish the impact of spray programs. A combined control intervention (figure 15) shows similar patterns and suggests that the single most effective intervention approach would be an early focus on larval control.

### Effects of interventions on predators

In this analysis the same conditions are considered as the preceding section but the potential impact of the control interventions on predators is also taken into account. In the case of the larval control intervention, 80% mortality in the predator population is assumed, as was observed by [20]. The predator pressure returns to its normal level after a time lag of 21 days (see Predator section).

To the best of our knowledge, no study has focused on predators on adult *Anopheles *within houses, but spiders in particular are thought to be very efficient in preying on mosquitoes. Here the impact of the destruction of these predators is investigated under an assumption that they represent an adult mosquito mortality of 5%. It is also assumed that the predator-pressure returns to its normal level after a time lag of 21 days.

**Figure 16:**
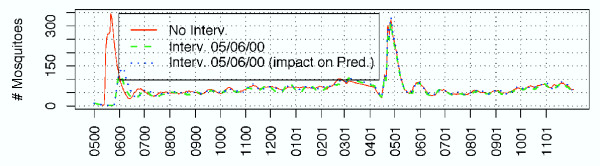
*An. gambiae *adult abundance with larval control intervention and predators

**Figure 17:**
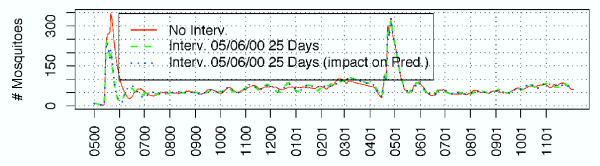
*An. gambiae *adult abundance with adult control intervention and predators

**Figure 18:**
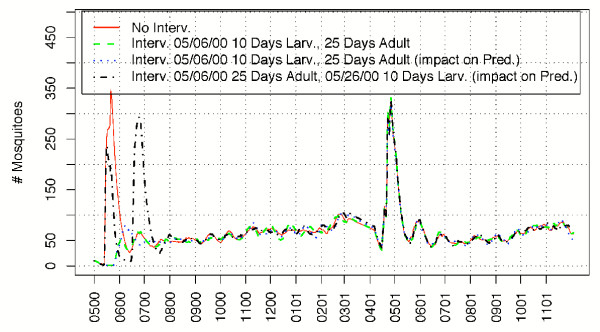
*An. gambiae *adult abundance with larval and adult control intervention and predators

Figures 16, 17 and 18 show the impact of predators on the vector population.

Figure 16 shows that the removal of predators has a big impact on the effect of a larval control intervention: the first peak is much less flattened, as it was in the previous section, and is displaced by about seven days.

The lack of predator pressure allows a much quicker reconstruction of the larval population.

For the adult control intervention, the curves in figure 17 show almost no differences. However, the half-life of the adult mosquito population increases by one day (from 4.6 to 5.7 days), which is of great epidemiological interest since this would increase the vectorial capacity by allowing more mosquitoes to become infectious.

Figure 18 considers the effects of a combined larval and adult control intervention for 10 and 25 days respectively and makes several points. First, the combined control intervention seems to have a stronger impact in terms of reducing the adult population. However, it was noted that the peak in adult abundance (with the predator simulation) is higher than the one without the predator simulation and also that there is a dramatic three-day increase in adult half-life (from 4.6 to 7.5 days). Furthermore, if the larval control intervention is delayed by 20 days, the consequences include not only the persistence of a fairly high first peak but also a higher second one. Therefore, such a model could be very important in helping to assess the optimal timing for vector control interventions.

## Discussion

This model integrates important mechanisms underlying *Anopheles *population dynamics in an explicit, transparent way. It focuses on five basic factors, two of them abiotic – temperature and moisture – and three biotic – nutrient competition, predation or death by disease, and dispersal.

Little of the published literature takes into account the effects of temperature on vector populations. It may be that temperature shows little fluctuation compared to countries with marked seasonality, but most African regions like Kenya exhibit temperature fluctuations ranging from 16°C to 35°C, which can be critical. Futhermore, temperature range is a key determinant for species dispersal and is, therefore, of high epidemiological importance: the species have different vectorial capacities and require different control programs.

Each parameter in equation 2 is individually related to the slopes of the curves for each stage of insect development (see Schoofield et al. [8]), and therefore may reflect a species' adaptation to different climates. Particularly, 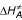, *ΔH*_*H *_and *ΔH*_*L*_, should reflect the sensitivity of each species to temperature changes in temperate, high and low temperature areas respectively, and thus could be highly informative. Many studies focus on vector breeding site characteristics, which the model addresses simply in terms of moisture. As yet no particular variables have been found to be crucial determinants of breeding site selection or success, but when these are determined, the model can implement them relatively easily. The transient patterns of breeding sites are taken into account as key determinants of predator and vector disease dynamics, however.

Nutrient competition is considered one of the major regulators of vector populations. Here the carrying capacity concept is used to allow both intra-and inter-species competition. Very few studies of vector predators and pathogens have been undertaken to date, but some literature suggests that this may also be an important determinant, so it has been incorporated accordingly. Little is known about *Anopheles *dispersal, though this is clearly a critical factor. Here simple random dispersal has been used, but it may be possible to implement a more sophisticated dispersal algorithm soon.

Thus, a basic tool has been developed for use by field workers and will be vastly improved by their efforts. First, more complete and precise data on *Anopheles *biology is needed: if nothing else, the model provides an organized view of the huge gaps in the existing information. A framework has been developed by exploiting what is available, but, at this point, far too many parameters and mechanisms involve arbitrary values or estimates.

Nonetheless, as an example, a vector population was simulated for a 20-month period, from May 1, 2000 to December 31, 2001, with meteorological data from Kilifi in Kenya and it was possible to roughly assess the sensitivity of vector population dynamics to four of the five basic factors – temperature, moisture, competition, and predation. The focus was on adult abundance curves.

Temperature is very important to the adult abundance curve and, particularly, to the occurrence of the initial peak after a drought period; this may be critical for control purposes. Moisture is a key determinant of particular high peaks that occur not only after a drought period but throughout the year for temporary breeding sites. These peaks were attributed to the lower larval mortality proceeding from lower predation and disease pressure.

These peaks may be of great epidemiological importance, in that they could bring malaria prevalence in humans above a threshold at which relatively high transmission could occur despite a low vector density. One concern with such large fluctuations is that the proportion of people susceptible may be very high at the beginning of the peak period. Furthermore, the earliest emergent adult mosquitoes may have a higher vectorial capacity; with almost no food competition, their weight is greater, which implies a longer life [26]. With different initial conditions, when high density competition induces longer development time, the occurrence of the first peak can be delayed by more than a week.

Preliminary results on species competition suggest the existence of competitive exclusion, i.e. the survival of only one species in a given habitat, which highlights the necessity of niche differentiation for species coexistence. The example also suggests that if insecticides impact populations of predators on *Anopheles*, the resulting de-regulation may backfire, producing a vicious cycle that leads to ever-increasing insecticide use. This further supports the argument that great improvements in our understanding of *Anopheles *ecology and population dynamics are needed.

The model is based on the data and knowledge currently available, and it can reproduce some broad, diverse patterns found in the field; its mechanisms and rules are explicit, and they allow us to provide detailed analyses and explanations of vector population dynamics. However, it requires considerable, continued application in the field to improve the data and our understanding of the underlying mechanisms. This is exactly the plan for subsequent research, to contribute to improved control of the scourge of malaria.

Table 9 shows the parameters in the most immediate need of field testing and measurement. However, with the default parameter setting, the model can currently be run by users with only:

1. A description of the geographical area with the pools and houses.

2. Climate information (temperature, precipitation, relative humidity) for the period considered.

## Conclusions

This model made it possible to structure existing knowledge of *Anopheles *vector population dynamics, and highlight crucial elements that are missing.

The data and other information currently available made it possible to build a model that can reproduce diverse patterns found in the field. It incorporates explicit mechanisms and rules that can provide detailed analyses and explanations, and thus is a tool to help the malaria research and intervention community gain a better understanding of vector dynamics.

The model should be greatly improved as more precise data and hypotheses become available and as it is applied in the field.

## Authors contributions

• JMD contributed conceptualisation and design of the model, main literature review and authorship of the paper.

• CM contributed conceptual and data input, review and comments.

• GK, BK, JB and JC contributed conceptual input, review and comments.

• JD, PB, HM, JG and AT contributed review and comments.

• FEM contributed the initial concept and general supervision.

All authors read and approved the manuscript.

## Figures and Tables

**Table 1:** Published or estimated (*) *An. gambiae *sensu lato immature stage developmental times (in days). The last point (**) is derived from the Jepson catenary curves.

**Temp. °C**	**Egg**	Larvae	Pupae	**Egg-Adult**	Species	Reference
15.1	2*	25.8*	2*	29.8	SL	[27]
16.3	2*	27.2*	2*	31.2	SL	[27]
18	1,5*	19.1*	1.5*	22.1	SL	[27]
18	1	21.3	1	23.3	ss	[28]
20	1	18.4	1	20.4	ss	[28]
21.9	1	12	1	14	SL	[29]
22	1	15.5	1	17.5	SS	[28]
22.1	1	14	1	16	SL	[29]
23	1	19	1	21	SL	[5]
23.5	1	10	1	12	SL	[29]
24		12.9			SS	[30]
24	1	11.5	1	12.5	ss	[28]
24.6	1	9	1	11	SL	[29]
25	1*	13.1*	1*	15.1	SL	[27]
25.3	1	10	1	12	SL	[5]
25.4	1	8	1	10	SL	[29]
25.5	1	13	2	16	SL	[5]
25.5	1	8	1	10	SL	[29]
26	1	11	1	13	SL	[5]
26	1	9.5	1	11.5	SS	[28]
26.8	1	8	1	10	SL	[29]
27		10.2			SS	[30]
27.2	1	9	1	11	SL	[29]
27.5	1	6	1	8	SL	[29]

**Table 2:** Published or estimated (*) *An. gambiae *sensu lato immature stage developmental times (in days). The last point (**) is derived from the Jepson catenary curves (continuing).

**Temp. °C**	**Egg**	Larvae	Pupae	**Egg-Adult**	Species	Reference
28		10.88			SS	[31]
28	1	7.8	1	9.8	SS	[28]
28.1	1	11	2	14	SL	[5]
28.2	1	7	1	9	SL	[29]
28.4	1	7	1	9	SL	[29]
28.4	1	7	1	9	SL	[29]
28.9	1	6 1		8	SL	[5]
28.9	1	6	1	8	SL	[29]
29.6	1	72		10	SL	[5]
30		8.3			SS	[30]
30	1	8	1	10	SS	[28]
30.7	1	5	1	7	SL	[29]
30.8	1	6 2		9	SL	[5]
30.8	1*	6.1*	2*	9.1	SL	[27]
31.2		7.9			SL	[32]
31.3	1	4	1	6	SL	[29]
31.4	1	8 1		10	SL	[5]
31.7	1	72		10	SL	[5]
32	1	8.2	1	10.2	SS	[28]
32.7	1	5	1	7	SL	[29]
32.8	1	6 1		8	SL	[5]
33.7	1	6	1	8	SL	[29]
37	1*	5.5*	1*	7.5**	SL	[5]

**Table 3:** *An. gambiae *developmental rate parameters.

	*ρ*_25°_C	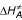	*ΔH*_*L*_	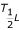	*ΔH*_*H*_	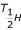
**Egg**	0.0413	1	-170644	288.8	1000000	313.3
**Larvae**	0.037	15684	-229902	286.4	822285	310.3
**Pupae**	0.034	1	-154394	313.8	554707	313.8
**Adult**	0.02	1000	-75371	293.1	388691	313.4

**Table 4:** Aestivation daily survival.

	Daily survival
Egg	0.8
Larvae	0.1
Pupae	0.3
Adult	0.96

**Table 5:** Vector weight parameters.

**Vector**	**Weight Min (mg)**	**Weight Max (mg)**
*An. gambiae*	0.236	0.383
*An. arabiensis*	0.33	0.45
*An. funestus*	0.2	0.33

**Table 6:** Proportion of death attributable to predation in *An. gambiae *larvae and pupae.

	**Stage duration (days)**	**With predators**	**Without predators**	**Attributable to Predators**
Larvae	9.98	90.9	79.58	11.34
Pupae	1.79	73.49	35.63	37.86
Total	11.77	97.6	86.85	0.11

**Table 7:** Water body characteristics

	sun exposure	coef water fill	water fix intake (*mm*)	water fix lost (*mm*)	max biomass density (*mg·m*^-2^)
Pool 1	1	4	0	0.01	30
Pool 2	1	7	0	0.02	60
Pool 3	0.7	4	0	0.028	70

**Table 8:** Impact of temperature on adult abundance.

**Temperature**	Mosquitoes
+2oC	47550.3
Normal	41449.9
-2oC	36199

**Table 9:** Parameters to define.

**Daily survival**
Egg aestivation survival
Adult aestivation survival
Adult aestivation trigger (relative humidity level/ factors combination)
Maximum larval biomass per surface unit
Egg survivorship
Larval predation mortality
Pupae predation mortality
